# Enhanced Patient Activation in Cancer Care Transitions: Protocol for a Randomized Controlled Trial of a Tailored Electronic Health Intervention for Men With Prostate Cancer

**DOI:** 10.2196/11625

**Published:** 2019-03-22

**Authors:** Mirjam Ekstedt, Kristina Schildmeijer, Camilla Wennerberg, Lina Nilsson, Carolina Wannheden, Amanda Hellström

**Affiliations:** 1 Department of Health and Caring Sciences Faculty of Health and Life Sciences Linnaeus University Kalmar Sweden; 2 Medical Management Centre Department of Learning, Informatics, Management and Ethics Karolinska Institutet Stockholm Sweden; 3 Department of Surgery Kalmar County Council Kalmar Sweden; 4 eHealth Institute, Department of medicine and optometry Faculty of Health and Life Sciences Linnaeus University Kalmar Sweden; 5 Department of Informatics Faculty of Technology Linnaeus University Kalmar Sweden

**Keywords:** medical informatics, eHealth, mHealth, motivation, patient activation, prostate cancer, self-management

## Abstract

**Background:**

Prostate cancer has increased in incidence worldwide and is the leading cause of cancer death in 24 countries. The most common treatment is radical prostatectomy. However, surgery is associated with postoperative complications such as urinary incontinence and sexual dysfunction, causing decreased quality of life. If survivors are encouraged to be more active in self-care management, the symptom burden may decrease and quality of life may improve. An electronic health (eHealth) intervention based on motivational behavioral theory has been developed for this purpose.

**Objective:**

This study aimed to compare the effectiveness of standard care in combination with a tailored eHealth and mobile health self-management support system, electronic Patient Activation in Treatment at Home (ePATH), with standard care of adverse effects of prostate cancer treatment (urinary incontinence and sexual functioning) in men undergoing radical prostatectomy. The secondary aim was to test the effect on patient activation, motivation, overall well-being, and health literacy over time in and between groups.

**Methods:**

A pragmatic multicenter, block-randomized controlled trial with 2 study arms, standard care (control) and eHealth-assisted standard care (intervention), for patients undergoing radical prostatectomy. For 80% power, a sample of 242 men will need to be recruited.

**Results:**

Recruitment started in January 2018 and is expected to be completed by August 2019. Data collection will be completed in August 2020. The first cross-sectional results from this trial are anticipated to be published in January 2020.

**Conclusions:**

With the increasing number of prostate cancer survivors, attention should be paid to rehabilitation, psychosocial care, and support for endurance of self-care to reduce suffering from adverse treatment effects, poor quality of life, and depression because of postoperative complications. This project may increase knowledge of how patients can be supported to feel involved in their care and returning to as normal a life as possible. The anticipated effects of ePATH could improve health outcomes for individuals and facilitate follow-up for health care professionals.

**Trial Registration:**

International Standard Randomised Controlled Trial Number: 18055968; http://www.isrctn.com/ISRCTN18055968 (Archived by WebCite at http://www.isrctn.com/ISRCTN18055968).

**International Registered Report Identifier (IRRID):**

DERR1-10.2196/11625

## Introduction

### Background

Prostate cancer is increasing in incidence worldwide, in both developed and developing countries, and it has been ranked as the eighth most common cause of cancer death globally. A growing and aging population must be considered as a contributing factor [1]. In Sweden, over 10,000 men are diagnosed with prostate cancer every year [2]. As novel diagnostic methods and treatment approaches have significantly improved cancer survival, the number of people living with disabilities after cancer treatment is also increasing [3]. The most common treatment is radical prostatectomy (RP), which shows >95% survival 15 years postsurgery [4]. Prostate cancer survivors commonly experience long-term consequences related to adverse effects of treatment, which can reduce quality of life. Specific adverse effects following local treatment (ie, RP and high-dose radiotherapy) include urinary leakage, loss of libido, and erectile dysfunction [5-7]. Further symptoms affecting functioning in everyday life, such as psychological distress, depression, fatigue, and muscular weakness, are prevalent [8], and an increased risk of suicide is evident in the first 12 months after diagnosis [9,10]. Thus, promoting support for management of long-term physical side effects for men with prostate cancer is of utmost importance.

In 2015, the Swedish government launched a national reform to standardize cancer care pathways with the goal of speeding up cancer treatment, increasing patient involvement and satisfaction with cancer care, and reducing waiting times and regional inequalities [11]. Each patient is assigned a contact nurse who will act as a primary caregiver contact throughout the treatment period and improve care coordination, availability, and patient participation throughout the care trajectory in line with the national reform. Yet, patients have reported unmet information needs along their cancer trajectories [11-13], especially lack of information about cancer treatment and its adverse effects, as well as a need for self-management support in the aftermath of surgery [14,15].

### Adverse Effects of Prostate Cancer Treatment

Health-related quality of life in men with prostate cancer is closely associated with urinary and sexual bother and dysfunction following prostate cancer treatment. Urinary incontinence (UI; incomplete emptying, frequent dysuria, urgency, weak stream, straining, or nocturia) is common, which may affect functioning in daily life by hindering return to work and participation in social and physical activities, events, or sports [6,7]. Sexual dysfunction after prostate cancer treatment is a multifaceted problem with UI in relation to sexual activities, various orgasmic disturbances, penile shortening, and *de novo* deformity. Concerns about possible recurrence of the cancer [7] are mixed with feelings of dissatisfaction with body image, reduced physical function, emotional lability, and decreased masculine self-esteem [5]. Thus, psychological factors in relation to sexuality as well as the importance of involving the partner should be acknowledged during the rehabilitation phase [16].

### Prostate Cancer Rehabilitation

Recovery after prostate cancer treatment focuses on urinary function, sexual function, and the return to daily life. Currently, pelvic floor muscle training (PFMT) is recommended for UI following prostate cancer treatment. However, previous research shows varying effects of PFMT [17], and there is controversy regarding whether postoperative PFMT is effective for achieving urinary continence. Only 2 of 21 studies from the Cochrane review by Campbell et al (2012) [17] showed that postoperative PFMT had a statistically significant benefit. Two systematic reviews [18,19] that investigated preoperative PFMT showed inconsistent results on common side effects, including erectile dysfunction and incontinence. Chang et al [18] found evidence to suggest that preoperative PFMT improves early but not long-term continence rates. The etiology of UI after RP is multifactorial, and the mechanisms for how PFMT improves postoperative continence in men have not been fully elucidated, which may explain the inconsistent results. Furthermore, adherence to PFMT exercises and optimal frequency and number of repetitions are still sparsely studied.

Rehabilitation of sexual function is often pharmacological. However, survivors of prostate cancer are less likely to gain a proper effect from medical treatment [20]. Medical treatment is directed toward erectile dysfunction, and oral treatment with phosphodiesterase-5 inhibitors (PDE5Is) is the first-line choice. Although treatment with sildenafil (a PDE5I) is often successful, discontinuation rates for medical treatments of erectile dysfunction, including sildenafil, are found to range from 50% to 60%. Thus, a high number of men choose not to continue using the medication despite treatment being efficacious [21,22]. The current literature suggests that penile rehabilitation should start as early as possible, preferably the day after surgery. This means that PDE5Is may be most effective if treatment is initiated as soon as the diagnosis and surgery dates are confirmed [23]. However, the problem is complex, and it is likely that medication alone is simply not enough [21]. Literature shows that hypoactive sexual desire following RP occurs in 60% to 80% of men. This could partly be because of a psychological impact of the cancer on mental health and body image [24]. Although there is a place for medical and surgical therapies in erectile function recovery and or preservation, psychological and sexual counseling may be equally important in sexual rehabilitation after RP [24]. Therefore, alternatives such as sexual therapy techniques (sexual communication and stimulation) have been suggested [25].

Exercise is increasingly seen as significant in prostate cancer rehabilitation as a strategy to enhance sexual function as well as improve feelings of masculinity and reduce the distress men experience after prostate cancer [26]. The national recommendations on physical activity in cancer are 20 min of endurance training daily, along with aerobic training or household work 1 hour per week [27]. A meta-analysis [28] shows that introducing exercise in the rehabilitation may reduce the loss of muscle mass, fatigue, and psychological morbidity that may arise from cancer treatment. First-degree evidence shows that exercise improves quality of life, fatigue, and body strength. A systematic meta-review suggests that exercise also has an effect on incontinence in men with prostate cancer [29], and PFMT together with exercise might have a positive effect on sexual activity [28,30]. Altogether, there is a growing body of literature suggesting that single interventions are not enough to address the complexity of adverse effects of prostate cancer treatment. Rather, a combination of multiple interventions, including PFMT, exercise, cognitive behavioral therapy, psychoeducation, and peer support, is suggested to be the most effective in decreasing distress and improving health-related quality of life [28,30,31].

### Web-Based and Mobile Electronic Health Apps

Electronic health (eHealth), referring to health services delivered or enhanced through the internet and related technologies [32], has the potential to meet patients’ needs for tailored information and provide person-centered self-management support and encouragement for sustained healthy behaviors [33]. Increasingly, medical and public health practices are supported by mobile devices (mobile health, mHealth) that allow for bidirectional communication or on-demand access to health services, extending the accessibility of support beyond temporal and physical boundaries [34,35]. Despite the opportunities provided by mHealth apps for remote support and communication and an abundance of apps on the market, only a few apps focus on supporting patients during cancer treatment and follow-up [36]. Existing studies suffer from poorly validated information, and there is still a lack of rigorous trials regarding quality of life and postoperative rehabilitation in prostate cancer. Ongoing studies in Canada [37], the United Kingdom [38], and Sweden [39] explore the effects on early detection or follow-up care of introducing e & mHealth prostate cancer care with a focus on increased patient participation and interaction. However, different theoretical frameworks and intervention strategies are used to approach this goal.

This protocol describes the third phase of the electronic Patient Activation in Treatment at Home (ePATH) project, which started in 2015, targeting the care trajectory of cancer patients. The first phase explored prostate cancer patients’ need for support, during cancer treatment and follow-up, that facilitates self-management activities and a proactive interaction with health care professionals [14]. The second phase encompassed development of the content and functionality of the eHealth- and mHealth-assisted self-management support system, ePATH, by applying user-centered design, testing and optimizing in iterative cycles to satisfy patient requirements, and adapt to the context [40,41].

The aim of the third phase was to compare the effectiveness of a tailored eHealth- and mHealth-assisted self-management support as a complement to standard care, with standard care. Primary outcomes are postoperative adverse effects (ie, urinary and sexual bother and function) in men undergoing RP. The secondary outcomes are physical activity, patient activation, motivation, health literacy, and overall well-being, which may be associated with the primary outcomes and have a mediating or confounding effect on the intervention.

The hypotheses of the study are the following:

1. The ePATH self-management support system will have a greater effect than standard care on patient-reported outcomes of UI and sexual bother and function at 1, 3, 6, and 12 months after RP.
2. The ePATH self-management support system will have a greater effect than standard care on (1) physical activity, (2) patient activation, (3) motivation, (4) health literacy, (5) overall health and well-being, and (6) endurance and adherence to self-care at 1, 3, 6, and 12 months after RP.

## Methods

### Design

This protocol describes a pragmatic multicenter block-randomized controlled trial with 2 study arms for patients undergoing RP: standard care (control arm) compared with standard care in combination with the ePATH self-management support system (intervention). A pragmatic trial design has been chosen to test whether the intervention (ePATH) works under normal conditions in routine clinical practice [42]. The protocol conforms to Standard Protocol Items: Recommendations for Interventional Trials guidelines (Table 1).

### Participants

All men diagnosed with prostate cancer at the study sites in Southeast Sweden where the chosen treatment is RP (open, laparoscopic, or robot-assisted) will be eligible. Inclusion criteria include being able to speak, read, and understand Swedish, having or being able to get a mobile BankID (the leading solution for electronic identification used in Sweden) for safe handling of personal information, having access to an email address, and being computer literate.

### Recruitment

**Figure 1:**
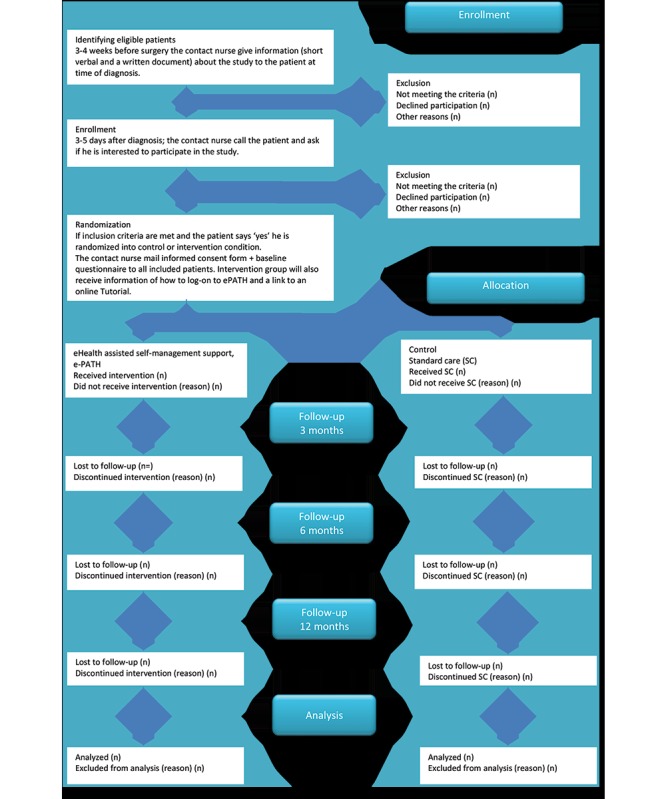
The CONSORT diagram shows how patients’ recruitment and flow through the randomized trial of two groups including attrition rate will be reported. (n) denotes the number of patients included and lost at each step.

Consecutive recruitment of patients will take place at 3 surgical and urology clinics in southeast Sweden*.* Eligible patients are to be identified by the contact nurses at the 3 study sites. In this study, the contact nurses will act as the communication channel among the researchers, the clinics, and the patients. The patients will receive both verbal and written information about the study in conjunction with diagnosis. Within 1 to 2 weeks, the contact nurses will contact each patient and ask about interest in participating in the study, and those who volunteer will submit their written consent form to the contact nurse (site B) or send their written consent in a prestamped, addressed envelope to 1 of the researchers (AH; site A and C; Figure 1). The researcher (AH) will send the Web-based baseline questionnaire to the patients. Afterward, the patients will be randomized to the 2 study arms. Patients randomized to the intervention group will receive an email informing them that an account has been created in ePATH. Patients randomized to the control group will receive an email telling them that no account has been set up (Figure 1). Due to the nature of the intervention, blinding of contact nurses or patients is not possible. None of the researchers are involved in patient care.

**Table 1:** Overview of timepoints, enrollment and assessments.

Study period		Timepoint						
		Enrollment: 4 weeks pre-RP^a^	Baseline: 2 weeks pre-RP	Discharge: 3-7 days post RP	Follow-up			
					1 month	3 months	6 months	12 months
**Enrollment**								
	Eligibility criteria: speak, read, and understand Swedish; Active email address; Planned RP surgery; Computer literacy; Access to computer, tablet, and mobile phone; Mobile BankID, or willing to apply for mobile BankID	X^b^	—^c^	—	—	—	—	—
	Information about the study	X	—	—	—	—	—	—
	Informed consent (written)	—	X	—	—	—	—	—
	Allocation: cluster randomization; Intervention: standard care+ePATH^d^; Control: standard care	—	X	—	—	—	—	—
	Journal data: cancer severity, length of hospital stay, type of surgery, postoperative complications	—	—	T_0_^e^	—	—	—	—
**Assessment**								
	Demographic data	—	T_0_	—	—	—	—	—
	Expanded Prostate Cancer Index Composite	—	T_0_	—	T_1_	T_2_	T_3_	T_4_
	Pelvic floor muscle training	—	—	—	T_0_	T_1_	T_2_	T_3_
	Physical rehabilitation	—	—	—	T_0_	T_1_	T_2_	T_3_
	Physical activity (SGPALS^f^)	—	T_0_	—	T_1_	T_2_	T_3_	T_4_
	Sexual rehabilitation	—	—	—	T_0_	T_1_	T_2_	T_3_
	Patient Health Questionnaire	—	T_0_	—	—	T_1_	T_2_	T_3_
	Needs Satisfaction Frustration Scale	—	T_0_	—	—	—	T_1_	T_2_
	Patient Activation Measure	—	T_0_	—	—	T_1_	T_2_	T_2_
	Cancer Behavior Inventory	—	T_0_	—	—	T_1_	T_2_	T_3_
	General health (RAND-1^g^)	—	T_0_	—	T_1_	T_2_	T_3_	T_4_
	Sleep Condition Indicator (short version)	—	T_0_	—	—	T_1_	T_2_	T_3_
	Fatigue Severity Scale	—	T_0_	—	—	T_1_	T_2_	T_3_
	Communicative and critical health literacy	—	T_0_	—	—	—	T_1_	T_2_
	Total questionnaire items	—	67	—	33	53	71	77

^a^RP: radical prostatectomy.

^b^X: procedure will be done.

^c^Not applicable.

^d^ePATH: electronic Patient Activation in Treatment at Home.

^e^T_0…4_: Time point for baseline (=0) or follow-up (=1-4) assessment.

^f^SGPALS: Saltin-Grimby Physical Activity Level Scale.

^g^RAND-1: 1 item from Veteran’s RAND 12-Item Health survey.

### Sample Size

Considering an effect size of 0.5 and a maximum of 4 domains (urinary, bowel, sexual, and hormonal) on the Expanded Prostate Cancer Index Composite with a 2-sided 5% significance level and a power of 80%, a sample size of 121 patients per group will be necessary given an anticipated dropout rate of 25% [43].

### Randomization

A computer-generated block randomization list will be produced by an independent statistician, who is not involved in the trial, to allocate participants to the 2 study arms. The statistician will retain information about block sizes and the randomization list for the duration of the study. The order will be concealed from the researchers through the use of sequentially numbered, opaque, sealed envelopes. The envelopes will be opened consecutively by 1 of the researchers (CWe) when patients have been included and allocated to the study arms. The researchers involved in the study will be blinded to block sizes.

### The Electronic Patient Activation in Treatment at Home Intervention

ePATH is conceptually based on a theoretical framework, self-determination theory [44], and the assumption that promoting autonomy, competence, and relatedness are factors that foster intrinsic motivation and engagement in sustained self-management. Patient activation in self-care entails understanding one’s own role in the care process, having the knowledge, skills, and confidence to perform the required behaviors [45], and creating the social support necessary for the initiation and maintenance of the desired behavior [44,46]. In ePATH, autonomy is supported by an engaged patient-nurse interaction, enabling patients to make a selection of self-care activities. Relatedness is supported through the message function, which will enable a 2-way communication with the contact nurse. Support for autonomy, competence, and relatedness in ePATH is expected to enhance patients’ motivation to engage in self-care.

ePATH is developed as a Web-based tool and also comprises a mobile app for mobile phones. The system makes use of national services for safe authentication for citizens (mobile BankID) and health care staff (e-legitimation, SITHS). ePATH provides access to tailored comprehensive information about diagnosis and treatment, a rationale as to why self-care is needed, health care contacts, a list of medications, and, finally, a set of self-care activities, with self-selected goals and ratings related to the activities.

ePATH contains functionality, enabling the patient to communicate with his or her health care contacts, report self-care activities (eg, PFMT or physical exercise), symptoms of relevance for diagnosis (eg, UI, sexual bother and functioning, distress, and fatigue), and follow-up on reported data over time. Notifications to exercise or take medication can be activated to provide the patient with support and reminders.

Self-care activities in ePATH focus on PFMT, psychosexual self-management, and exercise, as these activities are directed at the main symptoms after surgery. Self-care activities for UI include PFMT in the form of Kegel exercises (ie, contraction and relaxation of the pelvic floor muscles) 3 times per day to increase strength. A suggested physical exercise program including endurance training (jogging, running, etc) and resistance training (weight lifting, sit-ups, planks, etc) will be available. The intensity of exercise is determined based on the physical condition of the patient and can be assessed by the patient using the revolutions per minute Borg scale in the app; examples of suitable activities have been developed in collaboration with a physiotherapist [47-48]. For most men, sexual function is affected by the surgery. To increase the patients’ understanding of the postoperative situation and rehabilitation options, ePATH provides information about medical treatments and self-care activities for the sexual rehabilitation. There is also a diary function, where the men can write down their feelings, experiences, and thoughts related to sexual bother and function.

### Intervention Group

The intervention group is treated in accordance with the standard guidelines for RP at the clinic and the group has access to the ePATH self-management support system. Information about ePATH and how to navigate the app is provided in a written pamphlet. For those participants who so wish, a face-to-face meeting with the contact nurse or researcher may be arranged for practical guidance and training. Contact nurses at each of the 3 study sites have likewise been trained in how to navigate in the ePATH as well as in the protocol to ensure standardized follow-up.

At inclusion, the contact nurse and the patient review self-care activities and tailored goals of self-care. Patients have the possibility to evaluate and adjust goals and activities during the care trajectory through the message function in ePATH, over the phone, or in person during follow-up visits. Furthermore, the nurses check that patients are aware of the functions of ePATH: how to send messages, register activities performed and medications taken, make notes, and activate reminders.

### Control Group

**Figure 2:**
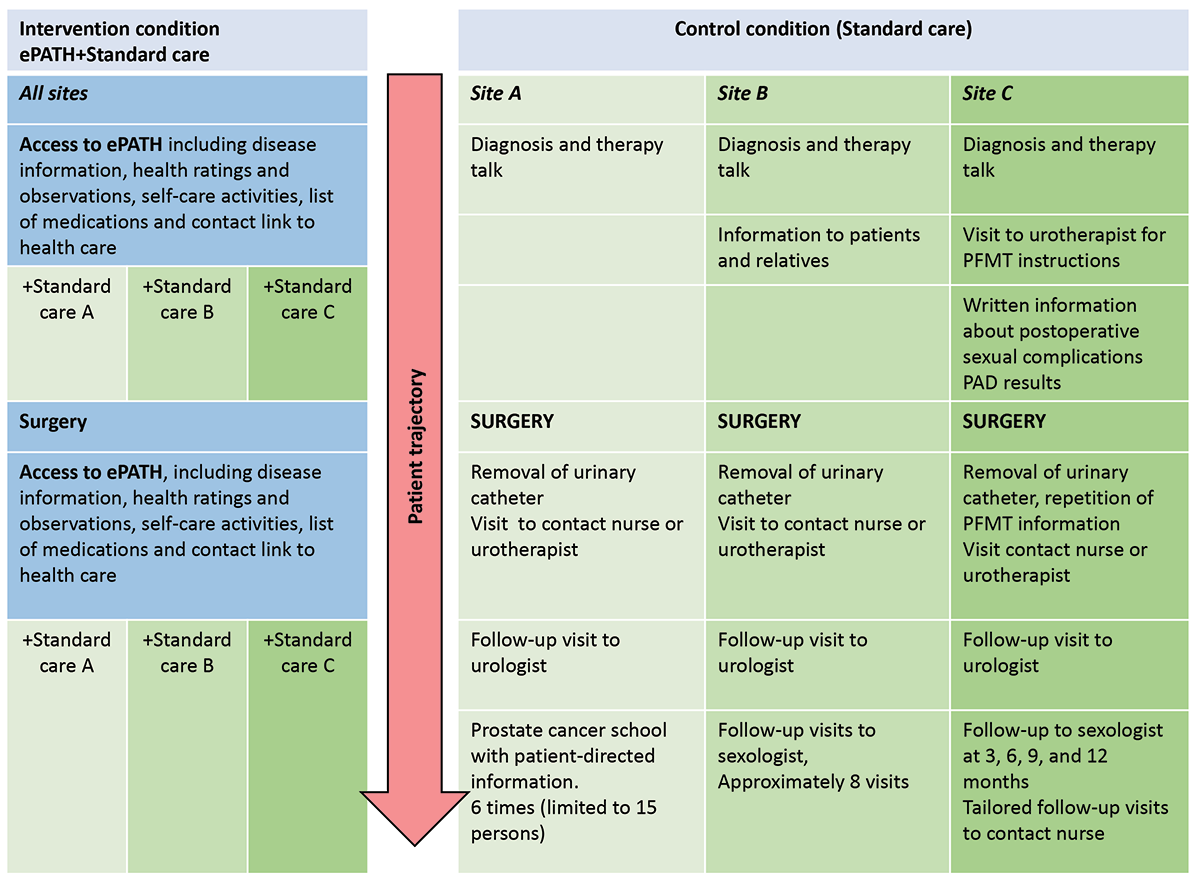
Patient trajectories at the 3 different study sites of the ePATH intervention.

Men randomized to the control group receive standard care following RP, with verbal and written information about PFMT (Kegel exercises), the diagnosis, the surgical procedure, and symptoms related to treatment (ie, urinary symptoms and affected sexual function). Follow-ups after discharge are in accordance with normal procedures at the clinics, with visits to an urotherapist or sexologist, a contact nurse, and a surgeon. As study sites are located in different counties, standard care is organized differently at each site (Figure 2).

### Outcome Measures

Participants in both study arms are asked to complete questionnaires at baseline and at 1, 3, 6, and 12 months after surgery. The contact nurses extract information on cancer severity (Gleason score), surgical procedure (open, laparoscopic, or robot-assisted), length of hospital stay, and possible postoperative complications that affected discharge from the patients’ medical records. A detailed time schedule of assessments and questionnaires is presented in Table 1.

At baseline, sociodemographic questions about age, marital status, educational level, and household income are included.

Primary outcomes are patients’ urinary and sexual bother and function, measured by use of the Expanded Prostate Cancer Index Composite-26 item, which contains 26 items to measure patient function and bother after prostate cancer treatment in urinary, bowel, sexual, and hormonal domains. Higher scores represent better health-related quality of life [43]. A total of 2 items concerning frequency of performed PFMT and support of sexual rehabilitation will also be included. See Textbox 1 for secondary outcomes.

### Analysis

Descriptive statistics will be presented with measures of central tendency and dispersion, as well as frequencies or percentages. The effectiveness of the intervention will be evaluated separately for the 2 hypotheses. As the trial involves repeated measures of data, a linear mixed model will be used to analyze the primary and secondary outcome measures. For ordinal outcome variables, a generalized estimating equation will be employed. The latter model will be used to evaluate the effect of the intervention on UI and sexual health at 1, 3, 6, and 12 months. An interaction term will be introduced into the model to examine the heterogeneity effect. A generalized linear model will be employed for intraindividual analysis of log data within the intervention group to evaluate associations between endurance and adherence to PFMT and postoperative outcome over time. We will perform residual analyses to assess model assumptions and goodness of fit. Assumption of normality will be checked using skewness and normal probability plot when necessary. An intention-to-treat analysis will be performed for the data. Patterns of missing data and dropout will be examined and, if necessary, multiple imputation will be used on the basis of the nature of the missing data. All statistical tests will be carried out at the 5% significance level (2-sided).

### Intervention Fidelity

To enable research to be implemented into clinical practice, it is important to report both internal and external contextual details on how the intervention has evolved during the research process. It is rare that a protocol for a randomized controlled trial is followed faithfully when delivering interventions outside laboratories or similar controlled environments. Therefore, it is necessary with flexible interventions that can be adapted to the target group and the unique circumstances of each clinical setting [57]. To describe those aspects as thoroughly as possible, we will follow the Template for Intervention Description and Replication checklist [58] (Table 2). We will carefully evaluate the intervention and its use to strengthen external validity through interviews with patients, retrieving log data from the ePATH app, and dropout analysis. We will also keep in close contact with the test clinics by phone, email, and visits throughout the study to make any necessary adaptions and modifications. All adjustments and contextual factors at the management level at the 3 study sites that may account for variations in implementation outcome will be documented and evaluated.

**Secondary outcomes.**

- Physical activity will be evaluated using a single-item measure of physical activity: the Saltin-Grimby Physical Activity Level Scale [47,48]. The respondent rates the time spent in physical activity per week on a 4-point scale: sedentary, some physical activity, regular physical activity and training, or regular hard physical training. An item about support received in physical rehabilitation will also be included.
- Patient activation is a latent trait that comprises knowledge, skills, motivation, and confidence a patient has regarding self-management of his or her illness, and it will be measured using the Patient Activation Measure-13 (PAM-13). PAM-13 contains 13 items that specifically measure patient active engagement in self-care [45]. Each item has 5 response categories with scores from 1 (strongly disagree) to 4 (strongly agree) and no score for not applicable.
- Motivation or self-determination, defined as perceived autonomy, social belonging, and belief in one’s own competence, will be measured using the Swedish short version of the Needs Satisfaction and Frustration Scale, comprising 6 items. Each item has 7 response options ranging from 1 (very often) to 7 (very seldom) [49].
- Health literacy will be measured using the Japanese Communicative and Critical Health literacy scale [50] comprising 5 items with 5 response options ranging from *never* to *always* [51]. A total score is calculated by collapsing the 5 response options into 3: never or seldom (1,000), sometimes (100), and often or always (1). These are then classified as lack of (1,000), problematic (100), and sufficient (1) health literacy, respectively.
- Overall, well-being will comprise the following measures:
  - The Sleep Condition Indicator Short Form*,* a screening tool for sleep difficulties with 2 items (score 0-4). A total score is calculated, with a lower score indicating poorer sleep [52].
  - The Patient Health Questionnaire-9, comprising 9 items (score 0-3) and representing depression as defined in Diagnostic and Statistical Manual of Mental Disorders-4th edition. A higher score indicates worse mental health. There is a tenth item covering the overall experienced difficulty of items 1 to 9 [53].
  - The Cancer Behavior Inventory-B *,* a measure of self-efficacy, comprising 12 items [54]. Confidence in one’s own capability is rated on a scale from 1 (not confident at all) to 7 (totally confident). A total score is calculated, and higher scores indicate greater coping efficacy.
  - The Fatigue Severity Scale, containing 9 items, with response alternatives from 1 (do not agree at all) to 7 (fully agree). A total score is calculated, and a higher score indicates greater fatigue [55].
  - General health, measured using a single item, with 5 response alternatives ranging from excellent (1) to poor (5), RAND-1 [56].
- Endurance in self-care and adherence to pelvic floor muscle training (PFMT) will be assessed through a single item asking how many times a day PFMT has been performed. For the intervention group, there is also a possibility to use ratings of practice in the ePATH. *Log data* will be retrieved from the ePATH, where the patients in the intervention group have the possibility to rate their self-care activities such as physical exercise (specified as type of activity, intensity, and time spent doing the activity), PFMT (frequency, duration), symptom burden, and health.

Patients’ usage of the ePATH self-management support system will be determined through extraction from the data logs to study user interactions with the different modules in the ePATH. Logistic regression will be performed to identify patterns of use, that is, associations between system use and patient characteristics. Such information is crucial in adapting the eHealth service to different patient groups.

Semistructured patient interviews will be conducted with a subsample of patients in the intervention group to provide deeper insight into how ePATH may support and promote adherence to self-care, strengthen patient health literacy, motivation, and the competences needed to manage self-care. We will also explore hindrances to self-care activities such as distress, anxiety, or insufficient or lack of support. Given the importance of understanding factors contributing to attrition, we will also interview patients who did not actively adapt to the ePATH. All interviews will be audio-recorded, transcribed verbatim, and analyzed using qualitative content analysis. These actions strengthen the external validity of the study, facilitating translation to other clinical settings.

### Ethical Considerations

The Regional Research Ethics Committee in Linköping (No 2016/484-31; 2017/512-32; 2018/147-32) has approved this study. For confidentiality, all communications and registrations of data in ePATH are transmitted through secure connections: Mobile BankID for patients and SITHS-login for care providers. All questionnaire and interview data will be anonymous and stored in accordance with the European Union legislation General Data Protection Regulation. Only the researchers will have access to data. Log data from the ePATH app will be stored in encrypted servers at Linnaeus University. Super administrators who have been responsible for the development and updates of ePATH will have access to the coding files of the app.

### Potential Harms

Criticism may be directed at the contact nurses if there are problems in the functioning and operability of ePATH. To avoid this situation, it will not only be emphasized that this is a research project where the views of the patients are valuable to improve ePATH but also that the contact nurses are merely users of the same eHealth tool: they are not responsible for it. If patients have criticisms, these should be addressed to the researchers. The intervention is unlikely to cause any harm to the participants, but there is a risk that patients may feel lost or deserted when the study ends. Therefore, ePATH will remain open for the intervention group patients to use after the 12 months have passed.

**Table 2:** Template for Intervention Description and Replication checklist for the electronic Patient Activation in Treatment at Home intervention.

TIDieR^a^ item	Description
1. Brief name	ePATH^b^
2. Why	To compare the effectiveness of a tailored eHealth^c^ intervention (ePATH) on the basis of self-determination theory with standard care. Primary outcome is postoperative symptoms in men undergoing radical prostatectomy. Secondary outcomes are patient activation, motivation, overall well-being, and health literacy over time in and between groups.
3. What materials	User manual (paper) and Web-based mobile app for both patients and health care staff (only available in Swedish).
4. What procedure	During use of ePATH, interactions with health care staff and registration of self-care activities are carried out on an individual basis.
5. Who provided	The intervention was developed as a codesign project with health care researchers, technicians, patients, and health care staff. Researchers with theoretical knowledge of self-determination theory and backgrounds in social and behavioral science, cancer care, and as registered nurses will provide the contact nurses with a training session of approximately 2½ hours. One such session will be held at each study clinic and include how to log in to and use ePATH, how to add targeted information, and how to communicate through the app. All study sites will receive a manual with a written, supplementary tutorial to ePATH, information about the study, the cornerstones of the intervention, the process of enrollment, and a checklist over the contact nurses’ responsibilities regarding enrollment and collecting data from the medical records of participants. The contact nurses also get to practice hands-on during introductory sessions. To ensure the patient’s ability to perform the intervention, that is, self-care activities, patients are provided with a user manual for ePATH, and the contact nurses will contact all patients in the intervention group to make sure that they can log in to ePATH.
6. How	Delivery of the intervention takes place on an individual basis, depending on the patients’ needs. The patients start using ePATH approximately 1 to 2 weeks before surgery, depending on how soon they get their surgery scheduled.
7. Where	On the Web and mobile phone in patient homes.
8. When and how much	The intervention is based on self-care activities and engagement of the patients in their own homes. The enactment of the intervention is monitored through ratings in the app, communication, and follow-up questionnaires. The patients themselves decide to what extent they wish to use ePATH.
9. Tailoring	Contact nurses, in their close communication with each patient, have the possibility to add self-care packages that are relevant for the individual. These packages can be changed and tailored over time.
10. Modification	Researchers have close communication with the contact nurses about adaptions, to facilitate the use of ePATH in the clinical reality. All adaptions and modifications (what, when, and why) are thoroughly documented by the researchers.
11. How well (planned)	Step-by-step inclusion documents have been developed together with each clinic to suit its routines. Inclusion of patients is followed continuously by the research team and there is an ongoing dialogue with the contact nurses about possible difficulties.
12. How well (actual)	Adherence by the patients (how well was the intervention received?), that is, how many performed the suggested self-care activities, how often and how long, is investigated through log data and individual interviews. Dropouts will be described in detail.

^a^TIDieR: Template for Intervention Description and Replication.

^b^ePATH: electronic Patient Activation in Treatment at Home.

^c^eHealth: electronic health.

### Data Management

The researchers are not involved in any of the care provided before, during, or after surgery. Documents and digital data will be stored in accordance with Swedish legislation on how to file research data, the Archives Act (Arkivlagen, SFS 1990:782). All data will be reported at a group level.

## Results

This project is supported by the Kamprad Family Foundation of Entrepreneurship Research and Charity (grant number 2015-0067), the Swedish Cancer Society (grant number CAN 2017/748), the Cancer Foundation in Kalmar County, and the Medical Research Council of Southeast Sweden (grant numbers FORSS-657211; FORSS-760131).

Recruitment started in January 2018 and is expected to be completed by August 2019. Data collection will be completed in August 2020. The first results from this trial are anticipated to be published in January 2020.

## Discussion

This project addresses the posttreatment challenges of prostate cancer, which create special requirements on customized rehabilitation and psychosocial support. Many RP patients suffer from poor quality of life because of postoperative complications. Erectile dysfunction and urinary leaks are common and may be difficult to talk about openly. With the number of men living with cancer expected to increase, major attention needs to be paid to rehabilitation and psychosocial care as well as the creation of tools to help patients return to as normal a life as possible. ePATH for those living with prostate cancer, their families, and health care professionals provides access to a digital tool that can provide individualized information, be a communication link, and support self-care and empowerment during the course of care and treatment. The project will also increase the knowledge of how patients can be supported in performing self-care and feeling involved in their own care. If the anticipated effects of ePATH are found, this could imply significantly improved health outcomes for individuals and may also facilitate follow-up for health care professionals.

## Abbreviations

eHealth: electronic health
ePATH: electronic Patient Activation in Treatment at Home
mHealth: mobile health
PAM-13: Patient Activation Measure-13
PDE5Is: phosphodiesterase-5 inhibitors
PFMT: pelvic floor muscle training
RAND: Research ANd Development, a non-profit corporation in the USA
RP: radical prostatectomy
SITHS: an e-legitimation through WebTrust Certificate Authority
UI: urinary incontinence
